# Maximum intensity projection aids in diagnosing acute appendicitis and mobile caecum: A case report and literature review

**DOI:** 10.4102/sajr.v25i1.2153

**Published:** 2021-07-28

**Authors:** Kakia A.F. Namugenyi, Ferdinand M. Oompie, Kasandji F. Kabambi

**Affiliations:** 1 Department of Surgery, Faculty of Health Sciences, Walter Sisulu University, Mthatha, South Africa; 2 Department of Radiology, Nelson Mandela Academic Hospital, Mthatha, South Africa

**Keywords:** maximum intensity projection, multi detector computed tomography, childhood, acute appendicitis, mobile caecum

## Abstract

Appendicitis is a common childhood condition requiring surgical intervention and delayed diagnosis can have serious consequences. This report describes the case of a child who presented with an acute abdomen and intestinal obstruction. Multidetector (MD) CT demonstrated a left-sided caecum and an inflamed appendix with a faecolith. Maximum intensity projection (MIP) post-processing was key in identifying the appendicular artery and determine the diagnosis. At surgery, however, a mobile caecum and the appendix were positioned on the right side.

## Introduction

Acute appendicitis is a common condition in childhood, but a left-sided appendicitis related to a mobile caecum is rare.^1^ Appendicitis is usually diagnosed on the basis of clinical presentation and laboratory results; however, an atypical presentation may pose a diagnostic dilemma. The position of the appendix varies considerably depending on the position of the caecum.^2^ In individuals with situs inversus, midgut malrotation or an unusually long appendix (more than 6.8 cm in children), the appendix may be found on the left side of the abdomen.^1,3,4^ There are a few case reports of CT scan findings demonstrating a perforated appendix in association with caecal redundancy.^2,5^

## Patient presentation

A 12-year-old boy presented with generalised abdominal pain, abdominal distension and vomiting for 1 week. There was history of intermittent abdominal pain with constipation, which was managed at a nearby primary healthcare unit. There were no known comorbidities and no history of previous surgery.

On clinical examination, the child was ill looking and febrile (38 °C) but fully conscious. The abdomen was distended with generalised tenderness and guarding. Biochemistry was normal except for a raised white cell count of 15 500 mm^3^ (normal range: 5000 mm^3^ – 10 000 mm^3^).

**FIGURE 1:**
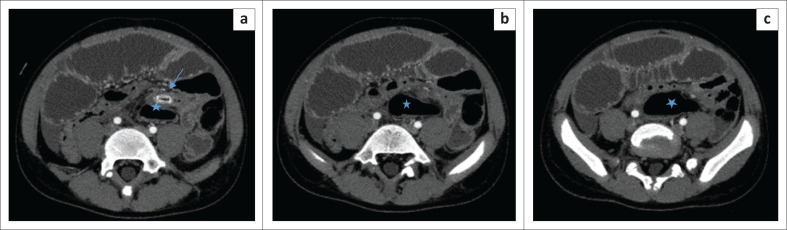
(a-c) (cephalad to caudad): A 12-year-old boy presented with abdominal distension, vomiting and a raised white cell count. Axial CT revealed the abnormal caecal position (blue star) in the left lower quadrant with an anterior cephalad thickened appendix containing an appendicolith (arrow) and adjacent fat stranding.

**FIGURE 2:**
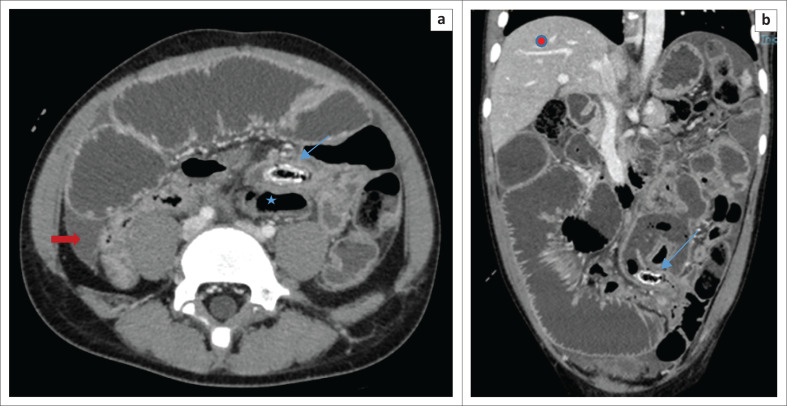
(a and b) Axial and coronal CT images demonstrating appendiceal wall thickening and enhancement with a faecolith (blue arrows) anterior and cephalad to the caecum (star). The right iliac fossa was unremarkable with fluid in the paracolic gutter (red arrow). Note the liver (red circle) is in the normal anatomic position.

**FIGURE 3:**
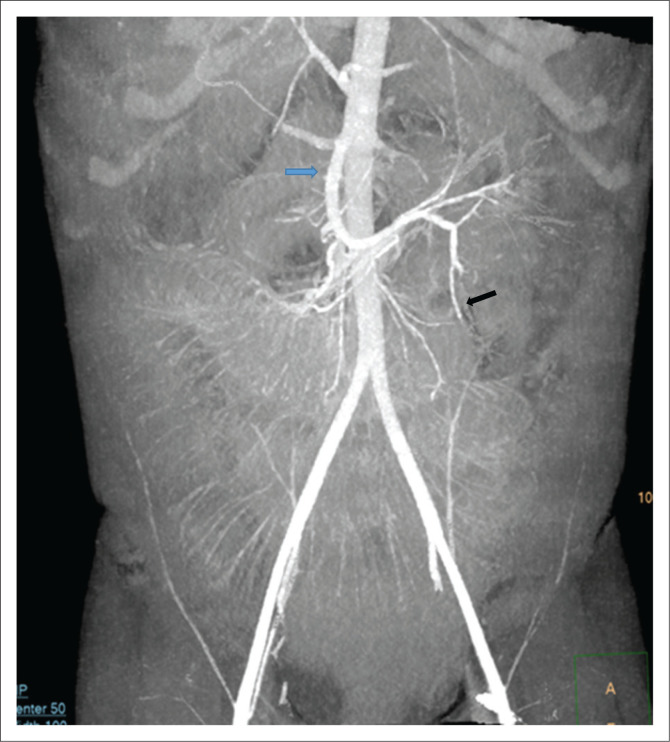
Coronal reconstructed maximum intensity projection image demonstrating the superior mesenteric artery (blue arrow) and its branches including the ileocolic (black arrow) from which the appendicular artery arises. The vessels are seen coursing to the left, corresponding to the position of the caecum and appendicolith. This is in contrast to the normal course towards the right lower quadrant.

Abdominal ultrasound demonstrated interloop free fluid and tenderness on probe compression. The appendix could not be identified because of overlying bowel gas in the distended bowel loops. Post-contrast-enhanced abdominal CT scan showed distended small bowel loops with multiple pelvic collections. The caecum was left sided (Figure 1a–c) with a 50 mm diameter collection in the left iliac fossa associated with a 7-mm long dense sausage-shaped structure, presumed to be the appendix with an appendicolith (Figure 2a and b). Differential diagnosis included a left-sided appendix with a faecolith, a foreign body complicated by perforated small bowel, infected mesenteric or duplication cyst and a Meckel’s diverticulitis with a stone. The radiologist sought to follow the appendicular artery, a branch of the ileocolic artery from the superior mesenteric artery (SMA) using maximum intensity projection (MIP) post-processing on a Philips IntelliSpace workstation (Philips Healthcare Netherlands B.V. Veenpluis 8.0, the Netherlands). The distal SMA was seen coursing towards the left in the region where the high-density sausage-shaped structure was seen (Figure 3). Interloop and right paracolic gutter fluid was present. A radiological diagnosis of a ruptured left-sided acute appendicitis probably due to a mobile caecum or an unusually long appendix was made.

Differentials remained as given here. There were no features of midgut malrotation or situs inversus. The referring surgeons were informed and an emergency laparotomy was performed.

The surgeons performed a midline incision extending from epigastrium to the suprapubic region. Multiple dilated friable small bowel loops were found. Accidental iatrogenic rupture of small bowel was primarily repaired. The dilated loops were decompressed and the caecum was found on a free mesentery on the right side.

The appendix was inflamed with an appendicolith at the base and a ruptured gangrenous tip. Appendectomy was performed and the specimen was sent for histology. Interloop collections were drained followed by peritoneal lavage. There were no adhesions. The patient was discharged after four days later, following an uneventful recovery.

Histopathology reported a 7 cm × 3 cm appendix with an occluded lumen and a faecolith.

## Discussion

A mobile caecum is a congenital abnormality with a defective right colonic mesenteric attachment at the lateral peritoneum because of agenesis of the caecal mesocolon.^6^ The estimated prevalence of a mobile caecum and ascending colon is 10% – 20%.^2,7,8^ During embryological development, a mobile caecum arises from failure of fusion of the colonic mesentery with the posterolateral peritoneum. This results in a free mesentery of the caecum and occasionally the right colon, allowing them to freely move to any part of the abdominal cavity. Occasionally, the caecum may rotate causing volvulus but most of the time it remains in the normal anatomic position.^7^

Mobile caecum and right colon are commonly present in children as mobile caecal syndrome.^9^ Patients with this syndrome may present with chronic intermittent abdominal pain, distension, flatulence, dyspareunia, caecal volvulus and partial or complete bowel obstruction.^7^

Left-sided acute appendicitis may be difficult to differentiate from an infected duplication, mesenteric or urachal cyst or a Meckel’s diverticulitis.^10^ Meckel’s diverticulum is the most common congenital anomaly of the gastrointestinal tract, present in 2% of the population. It is usually asymptomatic, but 4% – 40% of people with Meckel’s diverticulum may experience complications of diverticulitis, haemorrhage, intussusception, small-bowel obstruction, stone formation or neoplasm. A urachal cyst may become infected and present with abdominal pain, fever, nausea, vomiting, dysuria, voiding difficulty, epididymitis and orchitis.

Mesenteric cysts have an incidence of less than 1:100 000 cases in the general population. They may be located in the mesentery, peritoneum or retroperitoneum. Most are benign but there is a 3% incidence of malignancy.^11^ Patients with symptomatic mesenteric cysts present with abdominal pain and distension with cysts rarely becoming infected. Duplication cysts of the gastrointestinal system are rare and often associated with vertebral defects, anal atresia, cardiac defects, tracheo-esophageal fistula, renal anomalies and limb abnormalities (VACTERL anomalies), especially imperforate anus and hemivertebrae. If symptomatic, they present with abdominal distension, gastrointestinal obstruction or obstipation and caecal duplications, resulting in intussusception.^12^

The differential of a left-sided acute appendicitis may not be promptly established in the emergency setting and is often delayed because of atypical clinical signs.^1^ Delayed diagnosis may result in complications such as perforation, abscess formation, peritonitis, sepsis, bowel obstruction, infertility and death.^13^ Chest and abdominal radiographs may be helpful in excluding situs inversus with identification of the heart and gastric bubble in the correct anatomical position. Midgut malrotation may need contrasted upper gastrointestinal studies or cross-sectional imaging. Graded compression ultrasound plays a vital role in the diagnosis of acute appendicitis, especially in children. However, in the presence of bowel obstruction, this modality may be limited by bowel gas. Multidetector CT is the gold standard for imaging acute appendicitis.^14^ By following the intestinal segments sequentially from the stomach to the anus or vice versa, one is able to demonstrate the location of the caecum and ascending colon.

Multidetector CT also has the capability to assess the vasculature.^5,7^ One limitation of MDCT in acute appendicitis is the overlapping range in maximal appendiceal diameter between inflamed and non-inflamed appendices. The presence of fat stranding, abscesses and an appendicolith are valuable ancillary findings in confirming the diagnosis. In children with minimal abdominal fat, pericaecal fat stranding may not be present, limiting confidence in a definitive diagnosis. The current case demonstrated a left-sided caecum and faecolith associated with an abscess in the left iliac fossa (see Figure 1a–c). The presence of multiple dilated bowel loops and collapsed large bowel with interloop collections provided challenges in tracing the bowel. The MIP was very useful in helping to visualise the course of the appendicular vessels. The SMA was followed to the ileocolic and the appendicular artery (Figure 3). These vessels normally course towards the right iliac fossa. However, in our patient they deviated to the left where an abscess and the appendicolith were seen. This, combined with the left-sided caecum improved confidence in diagnosing appendicitis.

Maximum intensity projection software is available on most radiology workstations in South Africa. The MIP provides improved display of vascular maps, improving visualisation of most segments of the vessel including the intraparenchymal branches.^15^ Source images are used to display the maximum intensities in the voxels with selection of optimum slab thickness depending on the orientation of the vessels and the density of the adjacent structures. As MIP is not a 3D volume-rendered application, one of the pitfalls is the false interpretation of the relationship of the vessels and adjacent structures in the presence of high-intensity structures.^15,16,17^ This necessitates the user to adjust the display parameters to include the degree of opacification and slab thickness that correctly depicts the vasculature. The MIP requires substantial editing in most cases to clearly depict the anatomical display of the vessels. The quality of the final product may vary according to the experience of the user and the vendor.^15^

## Conclusion

This case report highlights MIP as a diagnostic problem-solving tool in the atypical presentation of a mobile caecum. Familiarity with this post-processing software is useful for advanced imaging application in clinical practice. This case report also increases awareness of the mobile caecum as an anatomic variant, which may result in an atypical presentation of acute appendicitis.
